# A HOOI-Based Fast Parameter Estimation Algorithm in UCA-UCFO Framework

**DOI:** 10.3390/s23249682

**Published:** 2023-12-07

**Authors:** Yuan Wang, Xianpeng Wang, Ting Su, Yuehao Guo, Xiang Lan

**Affiliations:** School of Information and Communication Engineering, Hainan University, Haikou 570228, China; wyuan@hainanu.edu.cn (Y.W.); suting4190@hainanu.edu.cn (T.S.); gyhao1996@hainanu.edu.cn (Y.G.); xlan@hainanu.edu.cn (X.L.)

**Keywords:** FDA-MIMO radar, HOOI, reduced-dimensionality MUSIC, parameter estimation

## Abstract

In this paper, we introduce a Reduced-Dimension Multiple-Signal Classification (RD-MUSIC) technique via Higher-Order Orthogonal Iteration (HOOI), which facilitates the estimation of the target range and angle for Frequency-Diverse Array Multiple-Input–Multiple-Output (FDA-MIMO) radars in the unfolded coprime array with unfolded coprime frequency offsets (UCA-UCFO) structure. The received signal undergoes tensor decomposition by the HOOI algorithm to get the core and factor matrices, then the 2D spectral function is built. The Lagrange multiplier method is used to obtain a one-dimensional spectral function, reducing complexity for estimating the direction of arrival (DOA). The vector of the transmitter is obtained by the partial derivatives of the Lagrangian function, and its rotational invariance facilitates target range estimation. The method demonstrates improved operation speed and decreased computational complexity with respect to the classic Higher-Order Singular-Value Decomposition (HOSVD) technique, and its effectiveness and superiority are confirmed by numerical simulations.

## 1. Introduction

A frequency-diverse array multiple-input–multiple-output (FDA-MIMO) radar is a nascent radar system that integrates frequency-diverse array and multiple-input–multiple-output technologies. The system introduces tiny frequency increments to generate range–angle-correlated transmission beams, which enable highly accurate and interference-resistant joint estimation of the range and angle of the target. Originally proposed by USA (United States of America) researchers in 2006 [1] and further developed by UK (United Kingdom) researchers in 2013 [2], the technique has a wide range of applications in aerospace, autonomous driving, IoT (Internet of Things), smart cities, and wireless communications to improve efficiency, security, and user experience [3,4,5,6,7]. An unfolded coprime array (UCA) with unfolded coprime frequency offset (UCFO) was proposed by researchers in 2022 [8]. Both the transmitter and receiver used a sparse uniform linear array (ULA) [9,10]. The signal was expanded by using negatively and positively biased portions of the transmitted signal by the framework. This increased signal bandwidth and array aperture and improved parameter estimation.

The parameter estimation algorithms for FDA-MIMO radars can be divided into two main categories: sparse-representation-based methods [11,12] and subspace-based methods [13,14,15,16]. The first one makes the most of the sparsity of the signal. It transforms parameter estimation into an optimization problem, which is solved by an iterative algorithm [17]. The latter method utilizes the orthogonality between the signal and noise subspaces. It constructs a two-dimensional spectral function, or it exploits the translation invariance property between array elements. Parameter estimation is accomplished by either finding spectral peaks or by solving for polynomial roots [18]. In later years, researchers began to investigate multidimensional data processing methods. One of the most popular is tensor parameter estimation, which uses tensors as the basic data structure to represent and process multidimensional data. Tensor parameter estimation algorithms mainly include Higher-Order Singular-Value Decomposition (HOSVD) [19,20,21] and Parallel Factor Analysis (PARAFAC) [22,23,24,25]. However, conventional tensor decomposition methods, such as HOSVD and PARAFAC, suffer from high computational complexity and memory consumption, especially for large-scale tensors.

In this paper, a tensor-based Higher-Order Orthogonal Iteration (HOOI) [26] is proposed for parameter estimation in a UCA-UCFO framework. HOOI has faster computational speed and better approximation accuracy compared to HOSVD and PARAFAC [27,28]. We apply tensor representation and processing to multidimensional data. Then, we use the HOOI algorithm for tensor decomposition of the received signal to acquire the core tensor and the orthogonal matrix. Next, they are employed to form a one-dimensional spatial spectral function to implement the Reduced-Dimension Multiple-Signal Classification (RD-MUSIC) method and conduct the direction angle estimation in low dimensions. Lastly, we derive the transmission steering vectors based on the characteristics of the transmission and the receiving steering vectors of the FDA-MIMO radar using a Lagrange multiplier. Relative to the traditional two-dimension MUSIC method, our method exhibits higher computational speed, lower computational complexity, better approximation accuracy, and improved estimation accuracy. The main contributions of the methodology put forward in this paper are as follows:

(1) The algorithm proposed in this paper is applied on the UCA-UCFO framework, where it is shown to be capable of parameter estimation accuracy improvement and has obvious advantages over ULA.
(2) The method proposed in this paper solves the problem of angle and distance estimation in the tensor domain as the tensor stores the inherent multidimensional structure of the signal model, which improves the accuracy of target parameter estimation.
(3) The HOOI algorithm used in this paper ensures the same accuracy as HOSVD while significantly improving the running speed, making the parameter estimation algorithm more suitable for real-time scenarios.

## 2. The Basics of Tensor and Signal Representation Using Tensors

### 2.1. Essentials of Tensors

First, we introduce some tensor basics relevant to this paper.

**Definition** **1** (Tensor unfolding of mode-n)**.** *Suppose $G\in C^{I_{1}\times I_{2}\cdots \times I_{N}}$ is an N-dimensional tensor, ${\left[G\right]}_{\left(n\right)}$ is defined as the n-mode tensor unfolding of G. The $(I_{1},I_{2},\ldots ,I_{N})\mathrm{th}$ element of G maps to the $\left(I_{n},J\right)$th element of G, where $J=1+\sum_{k=1,k\neq n}^{N}\left(I_{k}-1\right)J_{k}$ with $J_{k}=\prod_{m=1,m\neq n}^{k-1}I_{m}$.*

**Definition** **2** (Tensor matrix product)**.** *The mode-n product of a tensor $G\in R^{I_{1}\times I_{2}\times \ldots \times I_{N}}$ and a matrix $V\in R^{J\times I_{n}}$ is denoted by $H=G\times_{n}V$ with $H\in R^{I_{1}\times \cdots \times I_{n-1}\times J\times I_{n+1}\times \cdots \times I_{N}}$, and ${\left[H\right]}_{i_{1},i_{2},\ldots ,i_{n-1},j_{n},i_{n+1},\ldots ,i_{N}}=\sum_{i_{n}=1}^{I_{n}}{\left[G\right]}_{i_{1},i_{2},\ldots ,i_{n-1},i_{n},i_{n+1},\ldots ,i_{N}.{\left[V\right]}_{j_{n},i_{n}}}$.*

**Definition** **3** (HOSVD)**.** *HOSVD is a technique for higher-order tensor decomposition. It extends the Singular-Value Decomposition (SVD) of matrices to high-dimensional tensors and provides an efficient way to represent and analyze higher-order data. It is expression as:*

(1) $$G=H\times U_{1}\times_{2}U_{2}\times_{3}\cdots \times_{N}U_{N},$$

*where H is defined as the core tensor of G, the dimension of which is the same as for G. And $U_{n}$ represents the left singular vector of ${\left[G\right]}_{\left(n\right)}$.*

### 2.2. Signal Model Based on Tensors

**Figure 1:**
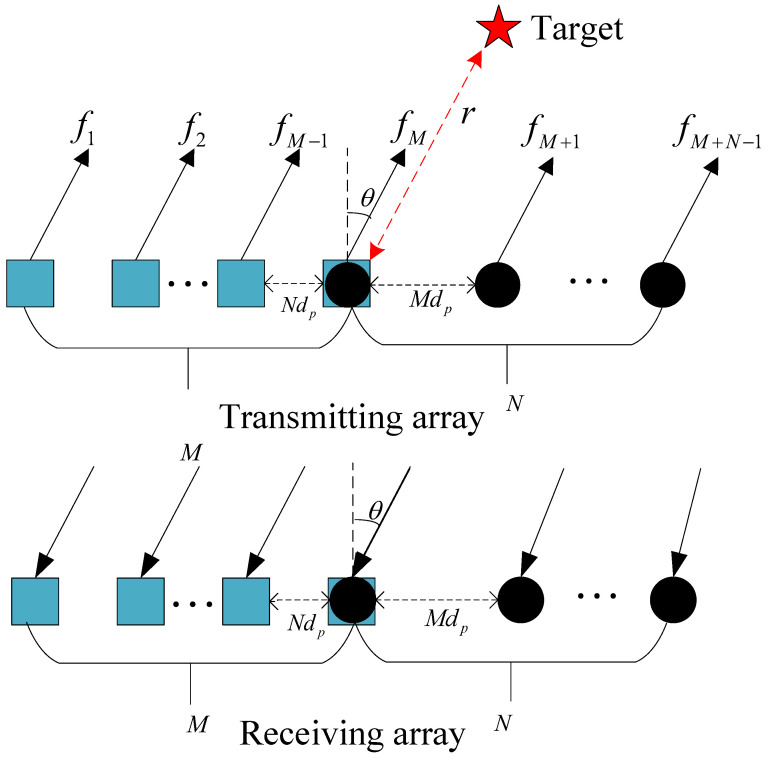
UCA-UCFO framework.

The paper [8] presents a novel FDA-MIMO radar framework called UCA-UCFO for explicit estimation of target angles and distances; all of the estimation work in this paper is based on this model. As shown in Figure 1, we set Q=M+N−1; the frequency of the *i*-th transmitting sensor is given by the formula

(2) $$\begin{array}{cc} & f_{i}=f_{0}+\left(i-M\right)R\Delta f.\\ & R=\left\{\begin{array}{cc} N, & 1\leq i\leq M\\ M, & M+1\leq i\leq Q \end{array}\right. \end{array}$$

Nd and Md are the array element spacings of subarray 1 and subarray 2, respectively, where *d* is the distance between adjacent elements, as shown below

(3) $$d≃c/2f_{0}=\lambda_{0}/2.$$

The transmit signal of the *i*-th transmitter unit is written as follows

(4) $$\begin{array}{c} s_{i}\left(t\right)=\varphi_{i}\left(t\right)e^{j2\pi ft}, \end{array}$$

where the range of variation of *t* is the radar pulse duration, and $\varphi_{i}\left(t\right)$ denotes the baseband waveform. Setting the time shift to *T*, provided that the waveforms are orthogonal to each other, yields

(5) $$\int_{0}^{T}\varphi_{i}\left(t\right)\varphi_{i}^{*}\left(t-\tau \right)e^{j\Delta f(i-i^{\prime })2\pi t}dt=\left\{\begin{array}{cc} 0,i\neq i^{\prime }, & \forall \tau \\ 1,i=i^{\prime }, & \forall \tau \end{array}\right.,$$

where τ is the time delay. The echo signal received by the *j*-th receiving sensor, assuming that electromagnetic waves propagate in space in an independent manner, can be characterized as

(6) $$\begin{array}{cc} y_{j}\left(t\right) & =\sum_{i=1}^{Q}\sigma \varphi_{i}\left(t-\tau_{i,T}-\tau_{j,R}\right)e^{j2\pi \left(f_{i}+f_{d}\right)\left(t-\tau_{i,T}-\tau_{j,R}\right)}, \end{array}$$

where σ and $f_{d}$ denote the radar cross section (RCS) and Doppler frequency, respectively. In addition, $\tau_{i,T}$ and $\tau_{j,R}$ transmit and receive the time delay, respectively.

The variable $\tau_{0}$ is defined as the total delay, which is equal to 2r/c. The structure of the *i*-th output signal received by the *j*-th receiver sensor after going through the *i*-th matched filter is as follows:

(7) $$\begin{array}{cc} y_{ji}\left(t\right) & =\sigma e^{j2\pi f_{d}\left(t-\tau_{0}\right)}e^{-j2\pi \frac{f_{i}}{c}2r}e^{-j2\pi (f_{i}+fd\left(\frac{d\sin\left(\theta \right)\left(i-M\right)\alpha_{i}}{c}+\frac{\sin\left(\theta \right)\left(j-M\right)\alpha_{j}d}{c}\right)}\\ & ≃\sigma e^{j2\pi f_{d}\left(t-\tau_{0}\right)}e^{-j4\pi \frac{f_{0}}{c}r}e^{-j4\pi \frac{\left(i-M\right)\alpha \epsilon_{i}\Delta f}{c}r}e^{-j2\pi f_{0}\left(\frac{\left(i-M\right)\alpha_{i}d\sin\left(\theta \right)}{c}+\frac{\left(j-M\right)\alpha_{j}d\sin\left(\theta \right)}{c}\right)}, \end{array}$$

where $f_{d}\ll f_{0}$, $\Delta f\ll f_{0}$; in addition, $\alpha_{i}\;\mathrm{and}\;\alpha_{j}$ are both N if they are within the scope of [1,M] or are both *M* if they are in the range of [M+1,Q]. The output of the *j*-th sensor of the receiver can be a signal that is visually represented below

(8) $$y_{j}\left(t\right)=e^{-j2\pi \frac{\left(1-M\right)a_{j}\sin\left(\theta \right)}{\lambda_{0}}d}\left[\begin{array}{c} e^{-4j\pi \frac{(1-M)N\Delta f}{c}r-j2\pi \frac{\left(1-M)N\sin(\theta \right)}{\theta_{0}}d}\\ \vdots \\ e^{-j4\pi \frac{-N\Delta f}{c}r-j2\pi \frac{-N\sin(\theta )}{\theta_{0}}d}\\ 1\\ e^{-j4\pi \frac{M\Delta f}{c}r-j2\pi \frac{M\sin(\theta )}{\theta_{0}}d}\\ \vdots \\ e^{-4j\pi \frac{(N-1)M\Delta f}{c}r-j2\pi \frac{\left(N-1)M\sin(\theta \right)}{\theta_{0}}d} \end{array}\right]s\left(t\right),$$

where $\begin{array}{cc} & y_{j}\left(t\right)\in C^{Q\times 1} \end{array}$ and $s\left(t\right)=\sigma e^{j2\pi f_{d}\left(t-\tau_{0}\right)}e^{-j4\pi f_{0}r/c}$. The outputs of all the matched filters are then stacked to form a vector, as shown below:

(9) $$x\left(t\right)=\left[\begin{array}{c} y_{1}^{T}\\ y_{2}^{T}\\ \vdots \\ y_{Q}^{T} \end{array}\right],$$

where $a_{t}\left(r,\theta \right)$ and $a_{r}\left(\theta \right)$ represent the transmitting and receiving steering vectors, respectively, and they both $\in C^{Q\times 1}$, i.e.,

(10) $$a_{t}\left(r,\theta \right)=\left[\begin{array}{c} e^{-j4\pi \frac{\Delta fN(1-M)}{c}r-j2\pi \frac{\sin(\theta )N(1-M)}{\lambda_{0}}d}\\ \vdots \\ \begin{array}{c} \begin{array}{c} e^{-j4\pi \frac{\Delta f(-N)}{c}r-j2\pi \frac{\sin(\theta )(-N)}{\lambda_{0}}d}\\ 1 \end{array}\\ e^{-j4\pi \frac{\Delta fM}{c}r-j2\pi \frac{\sin(\theta )M}{\lambda_{0}}d}\\ \vdots \end{array}\\ e^{-j4\pi \frac{\Delta fM(N-1)}{c}r-j2\pi \frac{\sin(\theta )M(N-1)}{\lambda_{0}}d} \end{array}\right],$$

(11) $$a_{r}\left(\theta \right)=\left[\begin{array}{c} e^{-j2\pi \frac{\sin(\theta )N(1-M)}{\lambda_{0}}d}\\ \vdots \\ \begin{array}{c} \begin{array}{c} e^{-j2\pi \frac{\sin(\theta )(-N)}{\lambda_{0}}d}\\ 1 \end{array}\\ e^{-j2\pi \frac{\sin(\theta )M}{\lambda_{0}}d}\\ \vdots \end{array}\\ e^{-j2\pi \frac{\sin(\theta )M(N-1)}{\lambda_{0}}d} \end{array}\right].$$

We consider *P* independent targets in the presence of noise range and angle $\left(r_{p},\theta_{p}\right)$, p=1,2,…,P in the far field. We can rewrite x(t) in (11) as below:

(12) $$\begin{array}{cc} x(t)= & \left[A_{1}\left(r_{1},\theta_{1}\right),\cdots ,A_{p}\left(r_{p},\theta_{p}\right)\right]s\left(t\right)+\eta \left(t\right)\\ & =A(r,\theta )s(t)+\eta (t) \end{array},$$

where $A_{p}\left(r_{p},\theta_{p}\right)$ is defined as $A_{p}\left(r_{p},\theta_{p}\right)=a_{r}\left(\theta_{p}\right)⊗a_{t}\left(r_{p},\theta_{p}\right)$, p=1,⋯,P, and $\eta \left(t\right)\in C^{Q^{2}\times 1}$ represents the additional white Gaussian noise matrix. The received signal matrix is generated subsequent to the reception of *J* snapshots, and it has the following form

(13) $$X=\left[A_{r}⊙A_{t}\right]S^{T}+N,$$

where $A_{r}\in C^{Q\times P}$, $A_{r}\in C^{Q\times P}$ and $S\in C^{J\times P}$. Using the tensor matrix expansion method of Definition 1, we can view the received data matrix in Equation (15) as sliced in the impulse dimension (third dimension). We construct the measurement tensor X by stacking the matrices $X\left(t_{j}\right)\left(j=1,2,\ldots ,J\right)$ along the snapshot way. The measurement tensor has dimensions Q×Q×J, which can be characterized as

(14) $${\left[X\right]}_{\left(3\right)}^{T}=X.$$

For the FDA-MIMO radar with a UCA-UCFO framework, we propose a tensor-based signal model. By applying multidimensional signals, i.e., tensors, for target localization, we can significantly improve the accuracy of target parameter estimation.

## 3. A HOOI Method for Parameters Estimation

### 3.1. Factor Matrices and Core Tensor Construction Based on the HOOI Algorithm

The HOOI algorithm consists of the following steps:

Step1: Suppose a *Z*-order tensor X; the factor matrix is $U_{Z}\in R^{I_{z}\times R_{z}},z=1,\cdots ,Z$ ($I_{z}$ is the size of the *z*-th tensor dimension). Let k=0, initialize the core tensor G as a zero tensor.
Step2: Let k=k+1, and for z=1,…,Z, perform the following operations
  (15) $$B_{k}\leftarrow X\times_{1}U_{1}^{T}\cdots \times_{z-1}U_{z-1}^{T}\times_{z+1}U_{z+1}^{T}\cdots \times_{Z}U_{Z}^{T}.$$
  Then compute the SVD value $B_{k(z)}=U\Sigma V^{T}$ of the mode-n extension of the tensor $B_{k}$ by determining the number of its principal singular values $R_{z}$, and then let $U_{z}\leftarrow U\left(:,1:R_{z}\right)$.
Step3: Compute the core tensor for the *k*-th iteration
  (16) $$G\leftarrow X\times_{1}U_{1}^{T}\times_{2}U_{2}^{T}\cdots \times_{Z}U_{Z}^{T}.$$
  Judge whether it converges or not: if the convergence condition is satisfied, then execute the next step; if the convergence criteria are not met or if the upper limit on the number of iterations has not been reached, go back to Step 2 and continue with the iterations.
Step4: Outputs the factor matrix $U_{1},U_{2},\ldots ,U_{Z}$ and the core tensor G.

The signal model allows the HOOI algorithm to reach the termination condition when only one iteration is performed. Based on the core tensor and factor matrix obtained from the HOOI, it can be obtained that

(17) $$X=G\times_{1}U_{1}\times_{2}U_{2}\times_{3}U_{3}.$$

Moreover, since we assume that the number of targets is *P*, the rank of X is *P*. By truncating the HOOI, the tensor-based subspace is obtained as

(18) $$X_{s}=G_{s}\times_{1}U_{s1}\times_{2}U_{s2},$$

where $G_{s}=X\times_{1}U_{s_{1}}^{H}\times_{2}U_{s_{2}}^{H}\times_{3}U_{s_{3}}^{H}$ denotes the truncated core tensor. The singular vectors of $U_{z}$ are sorted from largest to smallest by the corresponding singular values, and the first *P* singular vectors are extracted for composing $U_{sz}\left(z=1,2,3\right)$. Then $G_{s}$ is substituted into Equation (18); after simplifying, the result is written as follows:

(19) $$X_{s}=X\times_{1}\left(U_{s1}U_{s1}^{H}\right)\times_{2}\left(U_{s2}U_{s2}^{H}\right)\times_{3}U_{s3}^{H}.$$

From the above, we have the subspace based on a tensor, which can be characterized as

(20) $$U_{s}={\left[X_{s}\right]}_{\left(3\right)}^{T}=\left(U_{s2}U_{s2}^{H}⊗U_{s1}U_{s1}^{H}\right){\left[X\right]}_{\left(3\right)}^{T}U_{s3}^{*}.$$

By the definition of the tensor mode-n expansion, we can get the ${\left[X\right]}_{\left(3\right)}^{T}$, and this is substituted into Equation (20); the simplification of the formula is the following

(21) $$U_{s}=\left(U_{s2}U_{s2}^{H}⊗U_{s1}U_{s1}^{H}\right)V_{s3}^{*}\Lambda_{s3}.$$

So far, the estimation of the signal subspace tensor $U_{s}$ has been completed.

### 3.2. Angle of Arrival Estimation Using Reduced-Dimension MUSIC Algorithm via the Tensor-Based Method

The signal and noise subspaces being orthogonal is a fundamental principle of the MUSIC algorithm [29]. By orthogonal transformation, the signal subspace can be represented by an orthogonal column matrix. An expression for the noise subspace can be obtained using orthogonal projection as

(22) $$U_{noise}U_{noise}^{H}=I-U_{o}U_{o}^{H},$$

where $U_{o}$ is defined as the orthogonal basis of $U_{s}$, and we define $U_{orth}=U_{noise}U_{noise}^{H}$.

From the above description, the two-dimensional spectral function is given by

(23) $$t\left(\theta ,r\right)=\frac{1}{{\left[a_{r}\left(\theta \right)⊗a_{t}\left(\theta ,r\right)\right]}^{H}U_{orth}\left[a_{r}\left(\theta \right)⊗a_{t}\left(\theta ,r\right)\right]}.$$

Inspired by the literature [30], aiming at the characteristics of FDA-MIMO radar transceiver steering vectors, we investigate a ranging strategy aiming to reduce the computational complexity, which is elaborated by the following derivation process.

From the signal model, we can see that $a_{r}\left(\theta \right)$ and $a_{t}\left(\theta ,r\right)$ both satisfy $\in C^{(M+N-1)\times 1}$. Before simplifying $a_{r}\left(\theta \right)⊗a_{t}\left(\theta ,r\right)$, let us briefly introduce an arithmetic property about the Kronecker product as follows:

(24) (Q⊗W)(E⊗R)=QE⊗WR,

where ***Q***, ***W***, ***E***, and ***R*** are four matrices and there exists a matrix product of ***QW*** as well as ***ER***. In carrying out the derivation, we set $a=a_{r}\left(\theta \right)⊗a_{t}\left(\theta ,r\right)$; we express this equation as follows:

(25) $$a=\left[a_{r}\left(\theta \right)I_{1}\right]⊗\left[I_{Q}a_{t}\left(\theta ,r\right)\right].$$

Combined with the operational properties of the Kronecker product mentioned above, we can simplify Equation (23) as follows:

(26) $$a=\left[a_{r}\left(\theta \right)⊗I_{Q}\right]a_{t}\left(\theta ,r\right).$$

Through the above simplification process, the spectral function mentioned in Equation (23) can be simplified as follows:

(27) $$\begin{array}{cc} T(\theta ,r) & =\frac{1}{t(\theta ,r)}\\ & =a^{H}\left[I_{Q^{2}}-U_{o}U_{o}^{H}\right]a\\ \; & =a_{t}{\left(\theta ,r\right)}^{H}{\left[a_{r}\left(\theta \right)⊗I_{Q}\right]}^{H}U_{orth}\left[a_{r}\left(\theta \right)⊗I_{Q}\right]a_{t}\left(\theta ,r\right)\\ \; & =a_{t}{\left(\theta ,r\right)}^{H}T\left(\theta \right)a_{t}\left(\theta ,r\right), \end{array}$$

where $T\left(\theta \right)={\left[a_{r}\left(\theta \right)⊗I_{Q}\right]}^{H}U_{orth}\left[a_{r}\left(\theta \right)⊗I_{Q}\right]$. In order to eliminate the effect when the emission guidance vector $a_{t}$ is a zero matrix, the constraint is set, denoted as

(28) $$e^{H}a_{t}\left(\theta ,r\right)=1\;⟹a_{t}\left(\theta ,r\right)=\frac{1}{e^{H}},$$

where $e^{H}={\left[1,0,\cdots ,0\right]}^{T}\in C^{Q\times 1}$.

From Equation (27), the problem is essentially an extreme value problem with constraints. To solve the problem, we use the Lagrange multiplier method to construct the Lagrange function, and after simplifying and organizing, we get the following specific form:

(29) $$L\left(\theta ,r\right)=a_{t}{\left(\theta ,r\right)}^{H}T\left(\theta \right)a_{t}\left(\theta ,r\right)-\lambda \left(e^{H}a_{t}\left(\theta ,r\right)-1\right),$$

where λ denotes the Lagrange multiplier. Based on Lagrangian extreme conditions, the first-order partial derivatives of Equation (29) can be specified in the following way:

(30) $$2T\left(\theta \right)a_{t}\left(\theta ,r\right)-\lambda e=0.$$

Equation (30) associates the extreme value condition, i.e., by substituting Equation (28) into Equation (30). Accordingly, we can deduce that

(31) $$\begin{array}{cc} & \frac{\lambda }{2}T{\left(\theta \right)}^{-1}e=a_{t}\left(\theta ,r\right)=\frac{1}{e^{H}}\\ \Rightarrow & \lambda =\frac{2}{e^{H}T{\left(\theta \right)}^{-1}e}. \end{array}$$

After simplification, $a_{t}\left(\theta ,r\right)$ can be characterized as

(32) $$a_{t}\left(\theta ,r\right)=\frac{T{\left(\theta \right)}^{-1}e}{e^{H}T{\left(\theta \right)}^{-1}e}.$$

Substituting $a_{t}\left(\theta ,r\right)$ into Equation (23) yields the RD-MUSIC space spectral function, expressed in the following way:

(33) $$\begin{array}{cc} t(\theta ,r) & =\frac{1}{T(\theta ,r)}\\ & =\frac{1}{a_{t}{\left(\theta ,r\right)}^{H}T(\theta )a_{t}\left(\theta ,r\right)}\\ & =e^{H}T{\left(\theta \right)}^{-1}e. \end{array}$$

After reducing the dimension of the spectral function, we obtain a two-dimensional MUSIC spatial spectral function related only to the target angle. In this way, we can simplify the calculation and analysis of the spatial spectral function.

A spectral peak search is performed on the RD-MUSIC space spectral function, and since P targets are preset, the angles corresponding to the first P maximal peaks are chosen, i.e.,

(34) $$\begin{array}{cc} \hat{\theta } & =\arg\max t(\theta ,r)\\ & =\arg\max e^{H}T{\left(\theta \right)}^{-1}e. \end{array}$$

So far, the estimation of the target angle parameters ${\hat{\theta }}_{p}\left(p=1,2,\cdots ,P\right)$ has been completed.

### 3.3. Range Estimation

After estimating the angle of DOA, ${\hat{\theta }}_{p}\left(p=1,2,\cdots ,P\right)$ can be substituted into T(θ) to get T(θ). Then T(θ) and Equation (32) are used to reconstruct $a_{t}\left(\theta ,r\right)$. Based on the observation in Equation (10), we can find that $a_{t}\left(\theta ,r\right)$ has rotational invariance, which leads to the derivation that

(35) $$\Phi_{ANG}=a_{t}^{†}\left(1:M-1,p\right)a_{t}\left(2:M,p\right)\cdot \Phi_{AN},$$

(36) $$\Phi_{AMG}=a_{t}^{†}\left(M:M+N-2,p\right)a_{t}\left(M+1:end,p\right)\cdot \Phi_{AN},$$

where p=1,…,P, $\Phi_{AN}=e^{-j2\pi dN\sin(\hat{\theta })/\lambda }$, $\Phi_{AM}=e^{-j2\pi dM\sin(\hat{\theta })/\lambda }$, $\Phi_{AN}$ and $\Phi_{AM}$ eliminate the angular components, and the above $\Phi_{ANG}$ and $\Phi_{AMG}$ are the resulting diagonal matrices of eigenvalues associated with the range.

Define range estimate sets $S_{RN,p}$ and $S_{RM,p}$ as

(37) $$\begin{array}{cc} S_{RN,p} & =\left\{\frac{(\mathrm{angle}{\left(\mathrm{diag}\left({\hat{\Phi }}_{ANG}\right)\right)}_{p}^{T}+2K_{N}^{\prime }\pi )c}{-4\pi \Delta fN}\right\},K_{N}^{\prime }\in \left[0,N-1\right)\\ p & =1,2,\ldots ,P \end{array}$$

(38) $$\begin{array}{cc} S_{RM,p} & =\left\{\frac{(\mathrm{angle}{\left(\mathrm{diag}\left({\hat{\Phi }}_{AMG}\right)\right)}_{p}^{T}+2K_{M}^{\prime }\pi )c}{-4\pi \Delta fM}\right\},K_{M}^{\prime }\in \left[0,M-1\right)\\ p & =1,2,\ldots ,P \end{array}.$$

Comparing the actual range estimates and the ambiguous range estimates of a single target using $S_{RN,p}$ and $S_{RM,p}$, the formula for the actual range estimate is as follows:

(39) $$\begin{array}{cc} {\hat{r}}_{p} & =\frac{{\hat{r}}_{N,p}+{\hat{r}}_{M,p}}{2},\\ p & =1,2,\ldots ,P \end{array}$$

where ${\hat{r}}_{N,p}$ and ${\hat{r}}_{M,p}$ are the closest range estimations selected from $S_{RN,p}$ and $S_{RM,p}$, respectively.

By employing our proposed method, we have successfully achieved accurate estimation of target angle and range parameters. This proves the effectiveness and practicality of our algorithm.

## 4. Proposed Algorithm Performance Evaluation

### 4.1. Computational Complexity Analysis

In order to evaluate the efficiency of the proposed algorithm, we analyze its complexity in this subsection. The complexity analysis is shown as:

(1) The HOSVD computation complexity of $X\in C^{Q\times Q\times J}$ in Equation (17) is $O(Q^{2}J)$.
(2) Constructing a signal subspace in Equation (21) requires $O(Q^{4}+QK^{2}+Q^{2}J)$.
(3) Dimensionality reduction of a two-dimensional spectral Function (27) requires $O(Q^{2})$.
(4) The search for one-dimensional spectral peaks in Equation (34) requires $O(\frac{\Delta \theta }{r_{\theta }}Q^{3})$, where Δθ denotes the DOA search scope, and $r_{\theta }$ is the step size of the search.
(5) Estimating range using rotational invariance requires O(M+N+P(M+N−4)).

From the above analysis, it can be proved that the complexity of the proposed algorithm is $O(Q^{2}J+Q^{4}+QP^{2}+Q^{2}J+Q^{2}+\frac{\Delta \theta }{r_{\theta }}Q^{3}+M+N+P\left(M+N-4\right))$. For the 2D-MUSIC algorithm, its algorithmic complexity is $O(Q^{4}J+Q^{6}+90c\left(Q^{2}+1\right)\left(Q^{2}-P\right)/\Delta fa_{d}r_{d})$, where $a_{d}=0.002$ and $r_{d}=0.02$. Numerical analyses show that the conventional 2D-MUSIC algorithm suffers from high computational complexity, whereas the computational complexity of the algorithm presented in this paper is significantly reduced.

### 4.2. Cramér–Rao Bound (CRB)

From Equation (13), the received signal can be represented in the following way:

(40) $$X=\left[A_{r}⊙A_{t}\right]S^{T}+N.$$

The Fisher information matrix (FIM) can be characterized as

(41) $$CRB_{\theta }^{-1}=F_{\theta }=2J\xi Re\left\{{\left(\frac{\partial a(\theta ,r)}{\partial \theta }\right)}^{H}R_{N}^{-1}\left(\frac{\partial a(\theta ,r)}{\partial \theta }\right)\},\right.$$

(42) $$CRB_{r}^{-1}=F_{r}=2J\xi Re\left\{{\left(\frac{\partial a(\theta ,r)}{\partial r}\right)}^{H}R_{N}^{-1}\left(\frac{\partial a(\theta ,r)}{\partial r}\right)\right\},$$

where $R_{N}=\sigma^{2}I$, $\sigma^{2}$, ξ, and *L* represent the covariance matrix of the noise, the power of the noise, the power of the signal, and the number of snapshots, respectively.

The partial derivatives in Equations (41) and (42) are shown as

(43) $$\begin{array}{cc} \frac{\partial a(\theta_{p},r_{p})}{\partial \theta_{p}} & =\frac{\partial a_{r}\left(\theta_{p}\right)}{\partial \theta_{p}}⊗a_{t}\left(\theta_{p},r_{p}\right)+a_{r}\left(\theta_{p}\right)⊗\frac{\partial a_{t}\left(\theta_{p},r_{p}\right)}{\partial \theta_{p}}, \end{array}$$

(44) $$\frac{\partial a(\theta_{p},r_{p})}{\partial r_{p}}=a_{r}\left(\theta_{p}\right)⊗\frac{\partial a_{\iota }\left(\theta_{p},r_{p}\right)}{\partial r_{p}},$$

with

(45) $$\frac{\partial a_{r}\left(\theta_{p}\right)}{\partial \theta_{p}}=-j2\pi \frac{cos(\theta_{p})}{\lambda_{0}}dD_{cop}a_{r}\left(\theta_{p}\right),$$

(46) $$\frac{\partial a_{t}\left(\theta_{p},r_{p}\right)}{\partial \theta_{p}}=-j2\pi \frac{cos(\theta_{p})}{\lambda_{0}}dD_{cop}a_{t}\left(\theta_{p},r_{p}\right),$$

(47) $$\frac{\partial a_{t}\left(\theta_{p},r_{p}\right)}{\partial r_{p}}=-j4\pi \frac{\Delta f}{c}D_{cop}a_{t}\left(\theta_{p},r_{p}\right),$$

where $D_{cop}=diag\left[N(1-M),\cdots ,-2N,-N,0,M,2M,\cdots ,M(N-1)\right]$.

## 5. Simulation Results

In this part, we demonstrate the performance of the suggested algorithm by numerical simulations. We employ the Root Mean Square Error (RMSE) to assess the precision of angle and range estimation as below:

(48) $$RMSE_{r}=\sqrt{\frac{1}{P}\frac{1}{J}\sum_{p=1}^{P}\sum_{j=1}^{J}{\left({\hat{r}}_{p,j}-r_{p}\right)}^{2}},$$

(49) $$RMSE_{\theta }=\sqrt{\frac{1}{P}\frac{1}{J}\sum_{p=1}^{P}\sum_{j=1}^{J}{\left({\hat{\theta }}_{p,j}-\theta_{p}\right)}^{2}},$$

where ${\hat{r}}_{p,j}$ and ${\hat{\theta }}_{p,j}$ represent the estimates of range and angle, respectively, in the Monte Carlo experiment. In this paper, we examine and benchmark the holistic performance of the presented method with the ensuing methods: Estimation of Signal Parameters via Rotational Invariance Techniques (ESPRIT) [31], HOSVD-ESPRIT [32], and SUIT [8].

In all simulations, three non-coherent targets with (*θ*_1_, *r*_1_) = (−10.44°, 4000 m), (*θ*_2_, *r*_2_) = (5.22°, 5000 m) , and (*θ*_3_, *r*_3_) = (35.56°, 6000 m) are considered. Unless otherwise stated, all simulations are performed using the following operating conditions: M=6, N=5, Monte Carlo experimental number L=500, reference frequency $f_{0}=10$ GHz, the speed of light $c=3\times 10^{8}$ m/s, and according to Equation (3), the array element spacing d=0.015 m.

**Figure 2:**
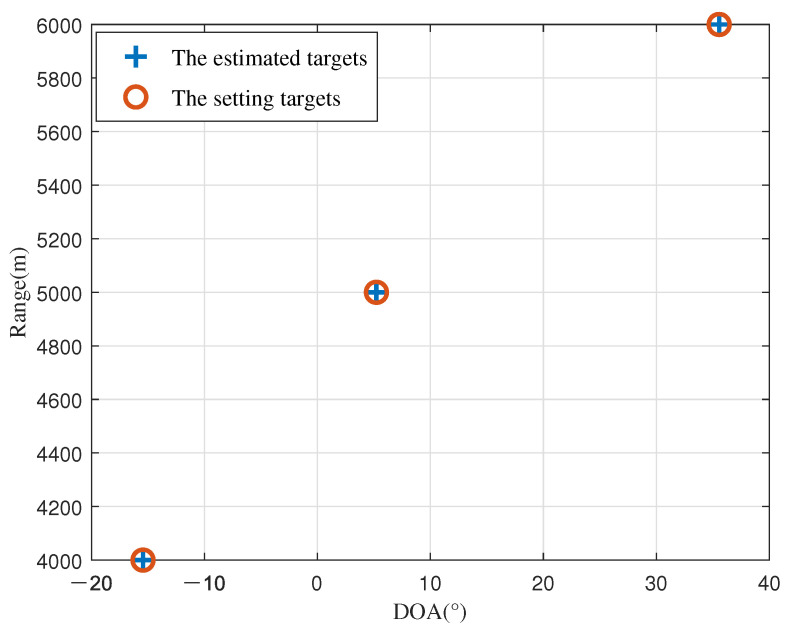
Estimation outcomes of the approach.

In the first experiment, we set the number of snapshots to *J* = 200 and the signal-to-noise ratio (SNR) to 20. The estimated angles and ranges closely align with the predefined values, as depicted in Figure 2. As illustrated in the figure, the experimental results confirm the reliability and performance of the method presented in this work.

**Figure 3:**
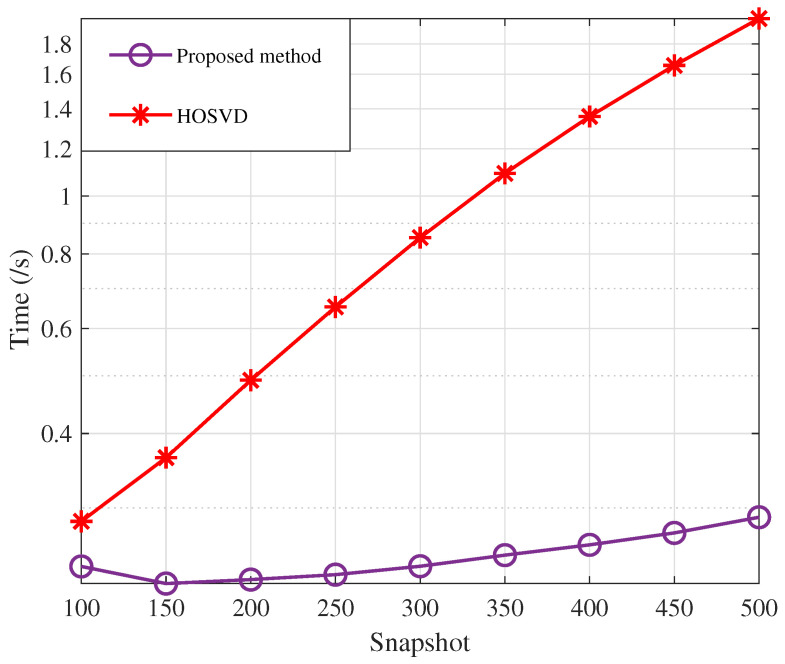
Algorithm runtime comparison.

In the second experiment, we configure SNR = 10 dB and examine how the algorithm execution time changes with the snapshot number. Figure 3 shows the factor matrix time cost for the core tensor and parameter estimation using HOOI and conventional HOSVD, respectively. Since the signal model in this paper allows HOOI to reach the termination condition at an iteration number of 1, whereas HOSVD requires singular-value decomposition of all modes, the running time of HOOI is much smaller than that of HOSVD.

**Figure 4:**
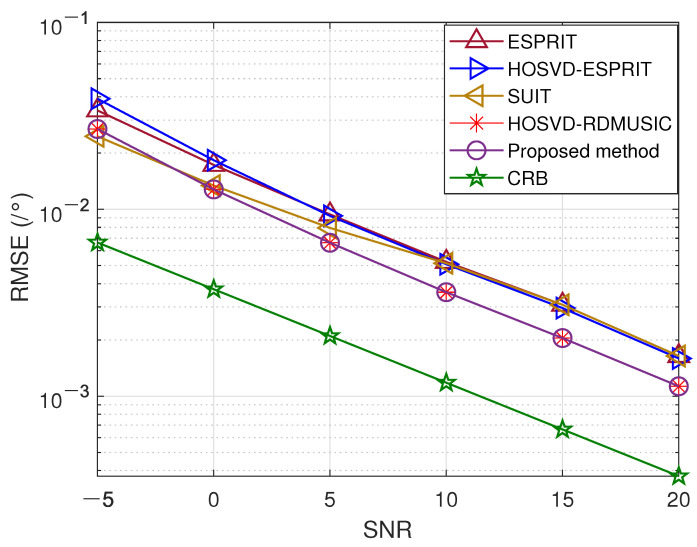
SNR versus DOA estimation error.

**Figure 5:**
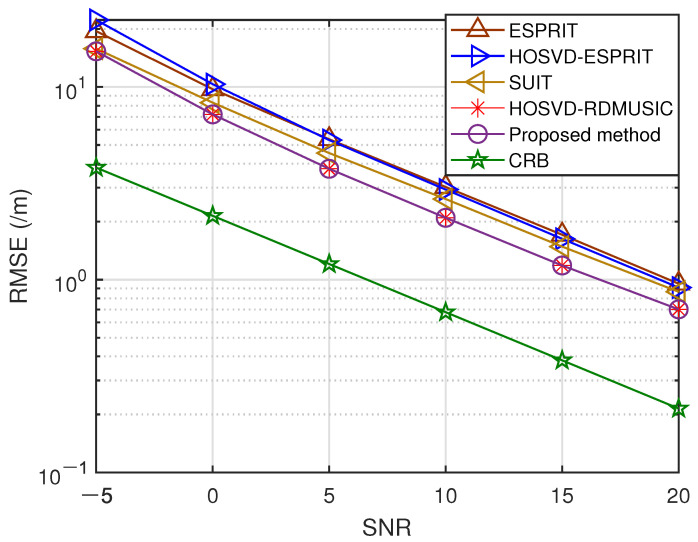
SNR versus range estimation error.

In the third experiment, we assess the effectiveness of the previously mentioned methods using different signal-to-noise ratios while J=200. The RMSEs for range and DOA are shown in Figure 4 and Figure 5, respectively. Our results indicate that the proposed method exhibits exceptional accuracy and stability, outperforming all other methods, including conventional HOSVD-RDMUSIC. This results from our presented method’s application of a multidimensional structure by tensors, which boosts the precision of target localization.

**Figure 6:**
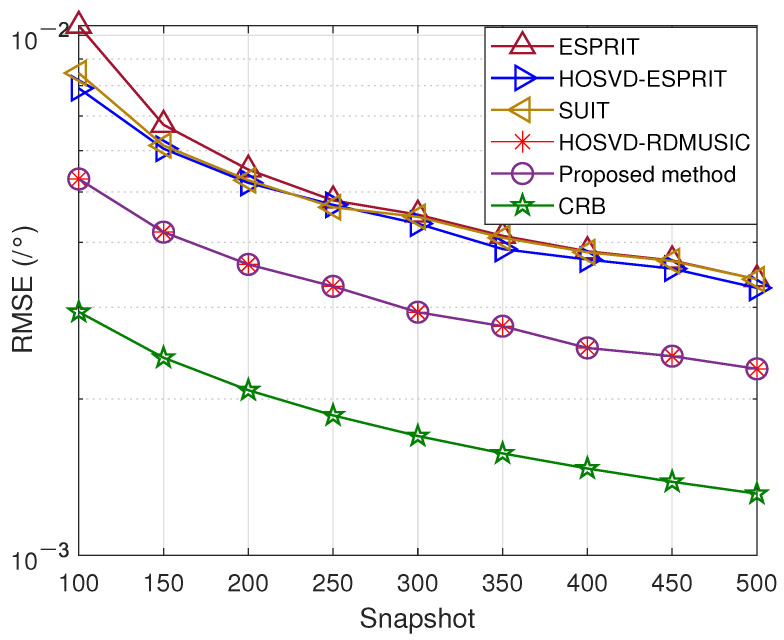
Snapshot number versus DOA estimation error.

**Figure 7:**
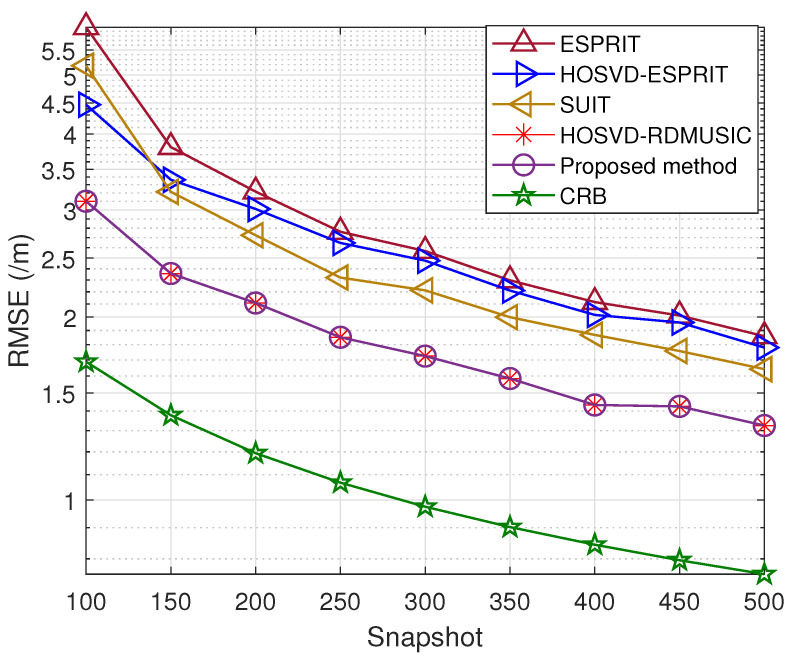
Snapshot number versus range estimation error.

In the fourth trial, Figure 6 and Figure 7 illustrate the DOA and range estimation RMSE obtained by the previously mentioned methods under different snapshot conditions at an SNR of 10 dB. Similarly, we also present the comparison method. It is clear that the curve of the RMSE for the suggested method is nearer to the Cramér–Rao Bound (CRB).

**Figure 8:**
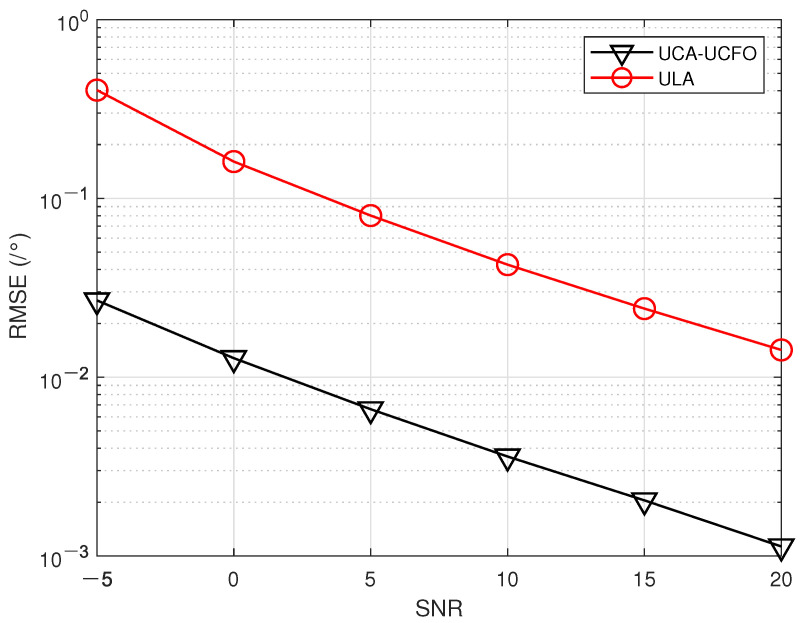
SNR ratio versus angle estimation error under different frameworks.

**Figure 9:**
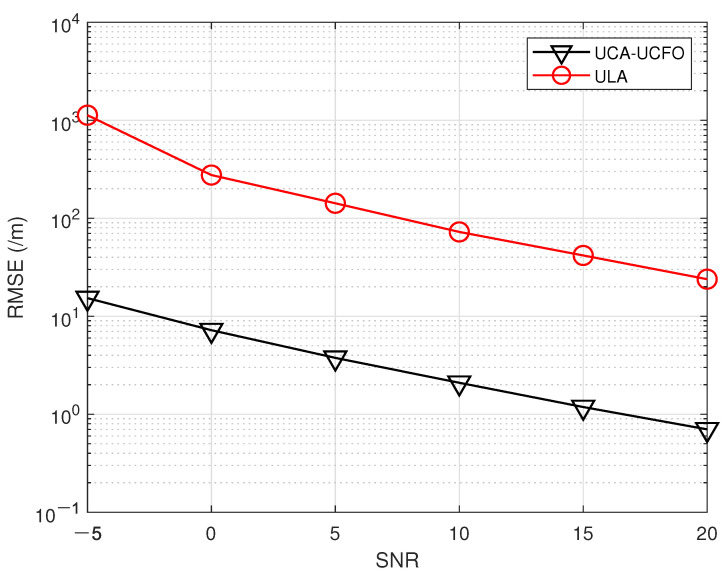
SNR versus range estimation error under different frameworks.

**Figure 10:**
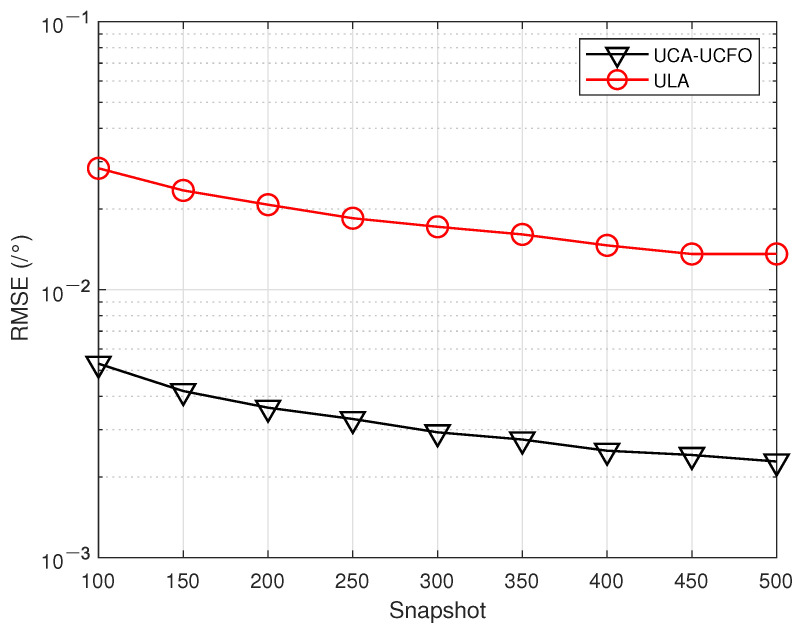
Snapshot number versus angle estimation error under different frameworks.

**Figure 11:**
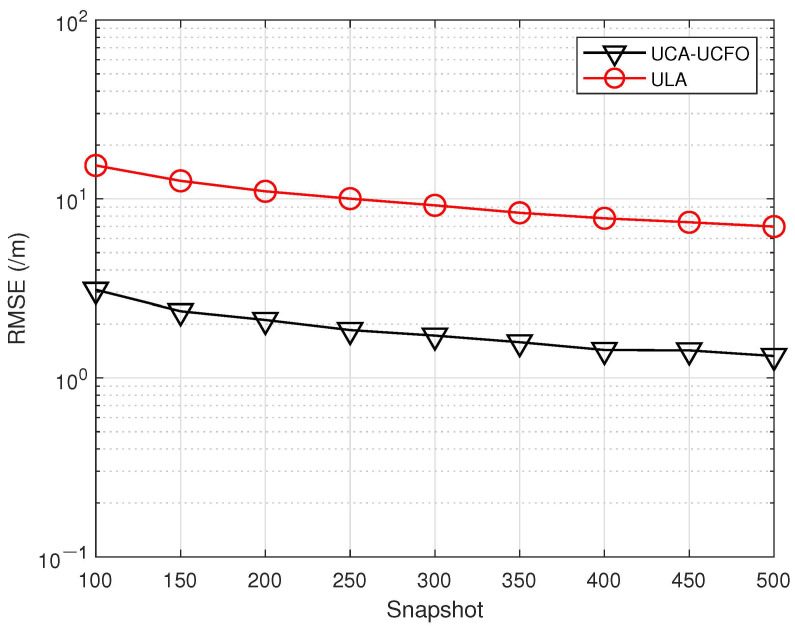
Snapshot number versus range estimation error under different frameworks.

In the fifth experiment, Figure 8, Figure 9, Figure 10 and Figure 11 illustrate a comparison of the estimation performance across different array geometries and frequency offset designs, comparing the UCA-UCFO framework used in this paper with the ULA arrays and utilizing the algorithm proposed in the paper for parameter estimation. As obtained from the figure, UCA-UCFO significantly improves the accuracy of angle and range estimation, outperforming ULA. The reason for this is that the UCA-UCFO framework is characterized by an unexpanded homogeneous number structure in both the angle and range domains, which gives it the best performance.

## 6. Conclusions

In this paper, a tensor-based Higher-Order Orthogonal Iteration (HOOI) is proposed for parameter estimation in a UCA-UCFO framework. The received signal is decomposed into a tensor by the HOOI algorithm to obtain the core matrix and factor matrix. Then, the 2D spectral function is built, and the dimensions are reduced to enable 1D spectral peak search and DOA estimation. After that, the transmission steering vector is obtained by taking the partial derivative of the Lagrangian function. Finally, its rotational invariance is exploited for target distance estimation. The suggested approach shows advantages: higher operation speed and reduced computational complexity relative to the conventional 2D MUSIC method, and its advantages are proved by the numerical analysis and simulation results.

## Data Availability

Data are contained within the article.

## Nomenclature

Y (bold Euler letters)	tensor
$C^{M\times N}$	M×N matrix set
Y (bold capital letters)	matrix
y (bold lowercase letter)	vector
${\left(\cdot \right)}^{*}$	conjugate
${\left(\cdot \right)}^{T}$	transpose
⨂	Kronecker product
⊙	Khatri-Rao product
${\left(\cdot \right)}^{H}$	conjugation-transpose
$I_{N}$	N×N elementary matrix
${\left(\cdot \right)}^{-1}$	inverse
${\left(\cdot \right)}^{†}$	pseudo-inverse
angle(·)	Extract the phase of the matrix
diag(·)	diagonalization of matrix
∘	Hadamard product
***0***_N_	N×N zero matrix
